# Telephone follow-up by nurse following total knee arthroplasty – protocol for a randomized clinical trial (NCT 01771315)

**DOI:** 10.1186/1472-6955-13-14

**Published:** 2014-05-21

**Authors:** Kirsten Szöts, Hanne Konradsen, Søren Solgaard, Birte Østergaard

**Affiliations:** 1 Department of Orthopaedic Surgery, University Hospital, Niels Andersens Vej 65, 2900 Hellerup, Denmark; 2 Research Unit, Gentofte University Hospital, Niels Andersens Vej 65, 2900 Hellerup, Denmark; 3 Department of Orthopaedic Surgery, Gentofte University Hospital, Niels Andersens Vej 65, 2900 Hellerup, Denmark; 4 Research Unit of Nursing, Institute of Clinical Research, Faculty of Health Sciences, University of Southern Denmark, Campusvej 55, 5230 Odense M, Denmark

**Keywords:** Total knee arthroplasty, Telephone follow-up, Health status, Self-efficacy

## Abstract

**Background:**

Due to shorter hospitalization, patients have to take responsibility for their rehabilitation period at a very early stage. The objective of this trial is to study the effects of two treatment schemes following total knee arthroplasty: conventional treatment following discharge from hospital and early follow-up by telephone consultations in addition to conventional treatment following discharge from hospital. The ultimate aim is to increase the effectiveness of the treatment by improving patients' health status, promote self-efficacy, and reduce the number of acute visits to the orthopaedic outpatient clinic during the rehabilitation period.

**Method/design:**

The design is a randomized un-blinded parallel group clinical trial conducted at the Department of Orthopaedic Surgery, Gentofte Hospital, the Capital Region of Denmark. In total, 116 patients will be allocated by an external randomization program to 2 groups: an intervention group following usual treatment after discharge supplemented by a nurse managed structured follow-up consultation conducted by telephone 4 and 14 days after discharge from hospital and a control group following treatment as usual. The consultations are structured by key subjects relevant to assess the health status according to the VIPS-model (the Swedish acronym for the concepts Well-being, Integrity, Prevention and Safety). The content of the consultations can vary according to the patients´ individual situations and needs. All consultations are conducted by the researcher responsible for the trial.

The effect is measured 1, 3, 6 and 12 months post-surgery. The primary outcome is self-reported physical function measured by The Western Ontario and McMaster Universities Arthritis Index. Secondary outcomes are self-reported health-related quality of life, general self-efficacy and the number of acute visits to the orthopaedic outpatient clinic.

**Discussion:**

The result of this trial is expected to provide new knowledge to support the development of targeted and effective follow-up after total knee arthroplasty in order to improve the patients´ health-related knowledge and skills of being able to take actively part in their illness and improve their health status.

**Trial registration:**

ClinicalTrials.gov: NCT01771315

## Background

### Introduction

The number of primary total knee arthroplasty (TKA) procedures is increasing and estimated to 1.4 million worldwide in 2015 [1]. In Denmark approximately 9000 TKA procedures are performed annually [2]. The main clinical indication for TKA is osteoarthritis [3] causing severe pain and substantial functional disabilities, leading to a decrease in health-related quality of life [4].

TKA is a common procedure that, despite of low level of mortality and complications, entails a severe surgical trauma and a protracted recovery [5].

The implementation of the fast-track programs for surgical patients has reduced the stay in hospital for TKA-patients, and the length of stay is now only around 3 days in several Danish surgical centres [6]. Commonly the patients are discharged to home and referred to physiotherapy in the community settings, with only one scheduled follow-up by the surgeon 3 months post-surgery.

During the early rehabilitation period TKA- patients have experienced several health problems especially physical ones [7]. In a survey conducted at Gentofte University Hospital in 2011, 96% out of 86 patients identified 1–7 physical postoperative health problems two to three weeks after undergoing TKA (unpublished observations by the researcher responsible for this trial). The health-related information given during the admission course has been difficult to transfer to the home settings [8], and following discharge the patients have needed further guidance [9]. Although the problems were apparent, the patients were reluctant to contact health professionals due to a belief that their problems were too insignificant to bother health care providers with [10]. During the rehabilitation period the experience of inadequate preparation for physical symptoms and psychological reactions, as well as unrealistic expectations to activity level have led to anxiety, depression and disappointment [11] to the extent of affecting the patients´ general health [12].

Self-efficacy is the degree of belief of having adequate action-oriented resources to control events affecting the everyday life successfully [13]. Self-efficacy is positively correlated with physical and mental aspects of health [14]. Self-efficacy is an important parameter during the rehabilitation period following TKA [15-17] by influencing physical function and mental health [16].

### Rationale for the study

Follow-up interventions after discharge of TKA patients have especially focused on the effect of various programs for physiotherapy. Internet based as well as home-based physiotherapy was assessed to be as effective as outpatient physiotherapy measured by physical function and health-related quality of life, respectively [18-21]. An intensive outpatient physiotherapy program additional to standard care (home-based exercise program) improved physical functional ability and health related quality of life compared to standard care [22]. Postoperative exercise is a highly prioritized part of the TKA treatment, aiming at improving the ability to practice daily activities immediately after surgery as well as maximizing the long term functional benefit of TKA [23]. However, the early rehabilitation period after TKA is characterized by a broad range of physical as well as mental symptoms as mentioned in the introduction.

A standardized follow-up program involving an exit video with role models, extra information through newsletters about the rehabilitation process, two telephone calls and weekly telephone hours was evaluated in a randomized clinical trial. The trial included 103 patients undergoing total knee and total hip arthroplasty eligible for a short stay (less than 6 days) in hospital and showed no effect on self-efficacy, social support and pain coping [24]. The program was not designed to provide individual counselling according to the patients´ personal circumstances and the progress during their rehabilitation period. In contrast a structured telephone follow-up focusing on individual care in regard to physical, social and mental aspects of the rehabilitation period had a positive effect on physical function, as well as general and mental health in 122 patients aged 65 years or older undergoing total hip arthroplasty [25]. It is assumed that it is possible to retrieve a corresponding positive effect for patients undergoing TKA.

### Aim

The aim of this trial is to evaluate the effect of a structured nurse managed telephone follow-up in the early rehabilitation period, based on the patient´s individual situation related to physical as well as psychosocial problems following TKA.

We hypothesize that telephone follow-up as a supplement to conventional treatment will improve health status and self-efficacy and reduce the number of acute clinical outpatient consultations after TKA compared to conventional treatment.

## Method/Design

**Figure 1:**
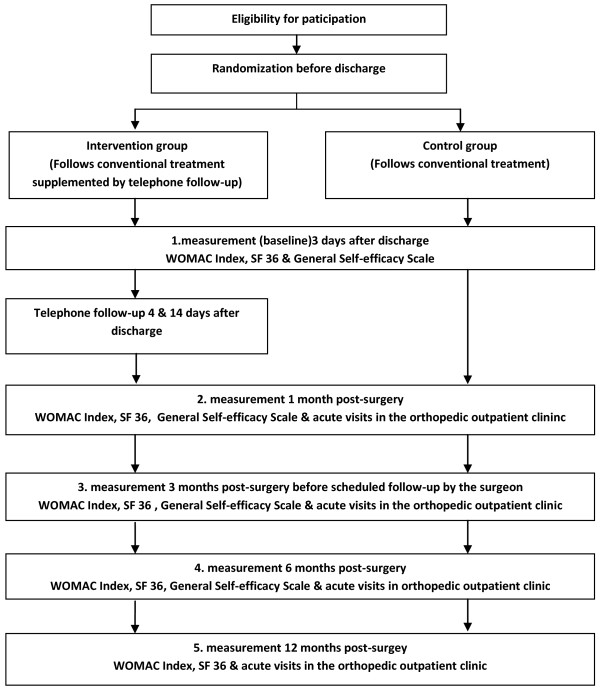
Flow-chart for the trial.

The study is designed as a randomized single centre parallel group clinical trial. The participants are allocated to two groups: an intervention group receiving telephone follow-up as supplement to conventional treatment and a control group that follows conventional treatment (see Figure 1).

### Primary outcome

In a 12 months period the effect of telephone follow-up is primarily assessed by a significant improvement of ≥ 12 points for the physical function score in the intervention group compared to the control group measured by the disease-specific Western Ontario and McMaster Universities Osteoarthritis (WOMAC) LK 3.1 Index [26].

### Secondary outcomes

Secondary analysis will assess the effect of telephone follow-up in the intervention group compared to the control group by change in pain and stiffness scores in WOMAC LK 3.1 Index, change in health-related quality of life measured by the Medical Outcomes Study Short Form (SF-36) [27], change in the General Self-Efficacy Scale score [28], and the number of acute visits to the orthopaedic outpatient clinic.

### Trial population and patient recruitment

All patients are recruited from Orthopaedic Department, Gentofte University Hospital – a medium sized hospital in the Capital Region of Denmark which performs approximately 600 primary TKA per year.

Patients who meet the inclusion criteria, and none of the exclusion criteria (see Table 1), are consecutively enrolled in the trial during admission. The researcher screens daily for eligibility of all patients scheduled for elective primary TKA. First day post-surgery potential participants are informed verbally and in writing about the aim and course of the trial with neutral use of words. The information is given by research assistants not involved in the trial and in their absence by the researcher.

**Table 1:** Eligibility criteria

**Inclusion criteria**	**Exclusion criteria**
● Primary first-time total knee arthroplasty due to osteoarthritis	● In terminal phase of another serious illness such as e.g. cancer with expected lifetime less than 6 months
● Age > 18 years	● Previous total hip arthroplasty
● Followed conventional course and discharged ≤ 4 days after surgery	
● Understand and talk Danish	
● Signed informed consent before randomization	

### Ethics

The Regional Committee on Health Research Ethics has assessed the trial and an approval is not required to initiate this trial. The regional Danish Protection Agency has approved the trial (no. 01839 GEH-2012-033).

The trial is conducted according to the latest Declaration of Helsinki [29]. All eligible patients are informed verbally and in writing about the aim and practical carrying out of the trial besides their rights as participants. All participants sign written informed consent forms prior to randomization.

All data will be handled with confidentiality, and the patients are ensured anonymity.

The trial is registered on CilinicalTrials.gov (NCT 01771315).

### Randomization and blinding

Patients are randomized 1:1 to the intervention group or the control group respectively. The randomization is performed centrally by a web-based randomization program and in blocks unknown to the investigator and other subjects involved in the study. The randomization is executed just before discharge of the patients from hospital.

### Conventional treatment

The patients are admitted to the orthopaedic department on the day of surgery or the evening before. Discharge is scheduled 2–3 days post-surgery based on the following criteria: able to manage personal hygiene by themselves, to walk with one or two crutches, and to climb stairs. The patients are referred to physiotherapy in the community settings, to removal of stitches or stables by their general practitioner, and to an outpatient consultation by the surgeon 3 months post-surgery.

All participants follow the conventional course of treatment for TKA-patients including the program for patient education pre-surgery conducted by a surgeon, physiotherapist, occupational therapist and a nurse, focusing on the surgical intervention, possible complications and risks, the admission course, and introduction to physical exercise training and equipment.

### Intervention

The participants allocated to the intervention group receive telephone follow-up 4 and 14 days post discharge in addition to conventional treatment. A uniform structure of the consultations is based on key subjects for nursing status defined by the VIPS-model (the Swedish acronym for the concepts Well-being, Integrity, Prevention and Safety). The VIPS-model is a process-oriented documentation model developed to generate structured general information about the patient’s condition, needs, desires, problems and resources relevant for nursing to provide adequate nursing interventions and evaluation of the outcomes [30,31]. The nursing status is based on predefined key subjects identified as relevant to assess the health status in the course of disease [31]. The telephone follow-up consultations are structured and contain the following key subjects: communication, cognition/development, breathing/circulation, nutrition, elimination, sleep, pain/perception, skin/tissue, sexuality/reproduction, activity and psychosocial /spirituality/culture. The themes are supplemented with specific issues relevant to health status after TKA in regard to treatment and observation of the wound and the operated limb, management of painkillers, and ability to exercise as recommended. However, the course of the consultations may vary due to the individual needs of the patients.

All interventions are conducted by the researcher responsible for this trial. The adherence of the protocol for the intervention is assessed by audiotaping consultations executed the first Wednesday in each month. Subsequently the records are checked by an external research assistant to ensure compliance with the protocol.

### Outcome measures

Outcome measures are collected at baseline defined by 3 days after discharge from hospital, and 1, 3, 6 and 12 months post-surgery. These data are obtained by self-administered questionnaires mailed to the patients, except for the questionnaire for baseline data, as the first questionnaires are handed to the patients just before discharge. The patients are contacted by phone once by research assistants if questionnaires are not received by the researcher 7 days after scheduled date for answering.

It is recommended to use a disease-specific health status measure supplemented by a generic measure of health-related quality of life to fully assess outcomes after TKA [32]. A combination of the WOMAC Index and SF-36 is the most frequently used combination [4] and will be used in this trial. Self-efficacy will be measured by the General Self-Efficacy Scale.

All questionnaires are validated and available in a Danish version.

### WOMAC Index LK 3.1

WOMAC is developed and recommended for evaluation of treatment effect of TKA [26,32]. The WOMAC Index includes 3 subscales: pain (7 items), stiffness (2 items) and physical function (17 items). The Likert scale version of the index will be used with the following description for all items: none, mild, moderate, severe, or extreme - corresponding to an ordinal scale of 0–4. The score in each subscale is standardized to a score of 0 to 100 with higher scores indicating more pain, stiffness and functional limitations.

Patients with osteoarthritis in the knee were involved during the development of the item inventory of the index in order to assess clinical relevance [26]. In comparison with several other instruments used to evaluate the consequences of osteoarthritis and generic measurements of health-related quality of life [26,32,33] the WOMAC Index has shown adequate properties in regard to validity.

The original, as well as European WOMAC Index Likert scale versions, have been tested as a patient completed questionnaire on TKA-patients pre- and post-surgery, presenting acceptable reliability concerning values for internal consistency (Crohnbach´s alpha y for pain .78 - .93; stiffness .75 - .93 and physical function .92 - .98) [26,34-36], and a test-retest reliability assessed by the intra-class correlation (pain .78 - 95, stiffness .67 - .90 and physical function .71 - .92) [34-36]. The ceiling and floor effect post-surgery were under 15% 6 months post-surgery, except for a ceiling effect at 15.79 for stiffness [37].

The responsiveness has been documented in several studies, and the mean changes at follow-ups 3 and 6 months post TKA-surgery were significant (p < 0.001) for all subscales [26,32,35].

The mean score of the subscale physical function 6 month post-surgery is estimated to 32 based on scores presented in the literature [38], allowing an improvement of 12 points as hypothesized.

### SF-36

The SF-36 is a generic measure of health status [39]. It yields an eight-scale profile of scores related to physical and mental dimensions of health based on the subscales: physical function (PF), role physical (RP), bodily pain (BP), general health (GH), vitality (VT), social functioning (SF), role emotional (RE), and mental health (MH). The dimensions represent the most frequently measured concepts in widely used health surveys, and they are the most affected by disease and treatment [40]. The answers in each subscale are converted to a score from 0 to 100, with higher scores indicating better health status.

SF-36 is recommended as a supplement to a disease specific measure to provide a broader insight into patients´ quality of life and allow comparison across conditions after TKA [32].

SF-36v2 in the acute form with a recall period of one week will be used in this trial.

### General self-efficacy scale

The uni-dimensional General Self-Efficacy Scale is developed to assess a general sense of perceived self-efficacy to predict coping with daily challenges and adaption after experiences of stressful life events. The scale can be applied after surgery as a mediator for health-related outcomes to assess changes in quality of life [28]. The scale encompasses 10 items, and the response to each item is made on a 4-points scale ranging from 1 = *not at all true* to 4 = *exactly true,* yielding a total score between 10 and 40. Higher score indicates higher level of self-efficacy [41].

### Data collection

From the medical records the following data are collected: age, gender, marital status, body mass index, ASA-group (American Society of Anesthesiologist physical status classification), co-morbidities, length of stay, and the code for the surgical intervention.

Further, the following patient-reported data are collected: level of education, occupational status, home care and nursing care at home, and inhibited physical function not caused by the TKA.

Additional patient-reported data about participation in the rehabilitation program, readmissions, and unscheduled contact with healthcare professionals (general practitioner, readmission and emergency department, doctor on call, the orthopaedic ward or the orthopaedic outpatient clinic) are collected as control for confounders.

Additionally, qualitative and quantitative data from the telephone follow-up consultations are documented in regard to duration as well as identified health problems and provided counselling related to the predefined themes structuring the intervention.

### Sample size

Calculation of the sample size is based on the primary outcome physical function in the WOMAC Index based on the smallest clinically relevant improvement, which is reported to 5.3, 11.8 and 20.4 points related to low, intermediate and high baseline score tertiles respectively [42]. A difference of a minimum of 12 points in favour of the intervention group compared to the control group during a period of 12 months post-surgery is assessed as a clinical relevant outcome in this trial and determined as basis for estimation of the sample size. The standard deviation for the entire population is estimated to 18 points. Sample size is calculated with the assumption of normally distributed data with α = 5% and 1-β = 90% and an equal number of patients in each group. It is estimated that 48 patients are needed in each group. With an expected drop out rate of 20% 58 patients have to be included in each group.

### Outcome analysis

Data will be entered in EpiData version 3.1. The statistical software STATA will be used for data analysis. Ratio-scaled data from both groups (intervention and control) will be compared by using parametric methods if data are normally distributed, and if not, nonparametric methods will be used. Nominal scale data will be compared by Chi-square test or by a 95% confidence interval when comparative measures. Categorical variables will be compared using the Pearson´s chi-square test and the Mann–Whitney test, and two-sided level of significance p < 0.05, if it is found optimal. For continuous data changes within the groups will be analysed by using a paired t-test. P < 0.05 will be considered as being statistically significant. Due to the repeated measures a logistic regression analysis will be performed.

All patients will be analysed in the groups to which they are randomly allocated according to intention to treat analysis. This analysis is primarily based on imputation of outcomes by carrying the last known outcome status forward and is supplemented by sensitivity analysis to examine the effect on the results.

### Results

The inclusion of participants was initiated in January 2013. In total, 50% of the intended participants are actually enrolled in the trial, and the inclusion is expected to be completed at the end of 2013.

## Discussion

Telephone follow-up is considered an inexpensive and easily organized intervention, and a good way to exchange information, provide health education and advice, manage symptoms and early recognition of complication, reassurance and quality aftercare [43]. A systematic review with focus on the effect of telephone follow-up initiated by health professionals and looking at physical and psychosocial outcomes did not lead to any conclusion [43]. It did though, give rise to a demand for further research, especially based on uniform and well described interventions, appropriate measurement instruments, and related to specific patient categories. According to our knowledge this is the first trial examining the effect of telephone follow-up consultations applied to patients undergoing TKA.

A well designed randomized trial is a reliable way to evaluate new treatment options by comparing them to accepted treatments [44]. A well performed randomization process ensures equal allocation to the intervention group and control group respectively, preventing selection of patients assessed suitable for the intervention. The consecutive enrolment and external web-based randomization with a block unknown to persons involved in this trial ensure random allocation and maintain concealment of the allocation sequence, as the patients and the persons enrolling patients cannot foresee the assignment [45]. Furthermore, the randomization process is executed just before discharge from hospital to avoid the outcome of the randomization to influence the counselling and information during hospitalization.

In non-pharmacological trials the care providers and the patients are frequently un-blinded [46]. The lack of blinding could affect the estimate of the treatment effect positively [47]. In this trial neither the patients nor the person conducting the consultations are blinded after the randomization, because it is impossible to hide whether the patients receive consultation or not. The participants who are assigned to receive a new treatment including extra follow-up may have favourable expectations, and those assigned to conventional treatment may be disappointed [48]. However, the patients have no previous experiences of the rehabilitation period after TKA and hereby no basis for comparison of any gain of the follow-up consultations with conventional treatment after being discharged following TKA. Furthermore, the control group has no knowledge of the content of the consultations, and contact between patients post discharge with opportunity to discuss benefits or disadvantages of follow-up consultations are unlikely. However, it cannot be excluded that the patients meet by chance at follow-up in the outpatient clinic.

Deviation from the protocol after randomization is expected, since some patients will withdraw, some may be unavailable at the times set for the consultations, and some may not return the questionnaires. Exclusion of the patients deviating from the protocol destroys the distribution of similar characteristics in the two groups and could influence the estimation of the effect of the intervention [49]. Intention to treat analysis will be performed including all randomized patients providing the least bias when comparing results between the two groups [50]. The drop-out rate can be expected to increase during the 12 months follow-up period and rapid response to unreturned questionnaires is executed by telephone calls to minimize the drop-out rate.

All interventions in this trial are conducted by the first author, reflecting the practice of that specific nurse and representing a positive attitude to the intervention, which could influence the outcome positively. The performance of the intervention will not reflect the intervention in an everyday clinical practice, which is influenced by the skills and attitudes of a range of nurses and the intervention needs to be tested in every day nursing practice to enhance generalizability. Random checks by audiotaping the intervention sessions and documentation in case report forms, assess treatment adherence essential to be able to appraise the feasibility and reproducibility of the intervention in clinical practice [51].

The effect of follow-up consultations by telephone after TKA has not previously been studied. Patient satisfaction with care related to information and contact with health professionals during stay in hospital was positively correlated with self-perceived health status up to 12 months after total hip and knee arthroplasty [52,53], and a long term effect of telephone follow-up consultations after TKA cannot be excluded. Due to timing and content of the intervention the effect is expected to be strongest and with the stated clinical relevant difference in scores between groups in the early recovery period.

Selection of patients enrolled in a trial may lead to problems with generalizability if the selected population differs in important ways to the more general one [44]. The practical value of a trial is supported by enrolling patients with characteristics that reflect the range and distribution of patients observed in clinical practice for a particular problem [54]. To enhance generalizability, all patients admitted are consecutively enrolled in this trial if they meet the inclusion criteria and none of the exclusion criteria. However, the sample in this trial will only represent the group of patients with a short stay in hospital, which minimizes the time for health-related counselling and information, but also increases the possibility of a positive outcome of the study.

### Perspective

This trial can be categorized as a pragmatic clinical trial with hypothesis and study design based on information needed to make decisions in the clinical practice. The trial addresses practical questions concerning benefits of the intervention as they will occur in routine clinical practice [54]. The results can provide new knowledge to support the development of targeted and effective follow-up after TKA, in order to improve the patients´ health-related knowledge and skills and enable the patients to take actively part in their handling of their illness and thereby improve health status.

## Competing interests

The authors have no competing interests.

## Authors’ contributions

All authors took part in designing the study, developing the protocol and drafting the manuscript. All authors have revised the manuscript critically and given their final approval of the version to be published.

## Pre-publication history

The pre-publication history for this paper can be accessed here:

http://www.biomedcentral.com/1472-6955/13/14/prepub
